# The first complete chloroplast genome of *Coptis chinensis* var. *brevisepala*, with implication for the phylogeny of Ranunculaceae

**DOI:** 10.1080/23802359.2018.1501308

**Published:** 2018-08-17

**Authors:** Reyim Mamut, Gulbar Yisilam, Hongwei Zhang, Faiza Hina, Zhaoping Yang

**Affiliations:** a College of Life Sciences and Technology, Xinjiang University, Urumqi, China;; b Key Laboratory of Conservation Biology for Endangered Wildlife of the Ministry of Education, College of Life Sciences, Zhejiang University, Hangzhou, China;; c Laboratory of Systematic & Evolutionary Botany and Biodiversity, College of Life Sciences, Zhejiang University, Hangzhou, China;; d College of Life Science, Xinjiang Normal University, Urumqi, China;; e Administration of Zhejiang Qingliangfeng National Natural Reserve, Hangzhou, China;; f College of Life Sciences, Tarim University, Alaer, China

**Keywords:** Adonideae, endangered plant, phylogenomics, plastome, Ranunculoideae

## Abstract

*Coptis* is one of the most important medicinal plant genera in eastern Asia. To better understand the evolution of this genus, the complete chloroplast genome of *C. chinensis* var. *brevisepala* was obtained by next-generation sequencing. The plastome of *C. chinensis* var. *brevisepala* is 155,426 bp in length, and consists of large (LSC, 84,488 bp) and small (SSC, 17,402bp) single-copy regions, separated by pair of inverted repeat regions (IRs, 26,768 bp). It harbours 111 unique genes, including 78 protein-coding genes, 29 transfer RNA genes, and four ribosomal RNA genes. *Rps19* and *ycf1* were pseudogenized due to incomplete duplication in IR regions. The nucleotide composition is asymmetric (30.5% A, 19.4% C, 18.7% G, and 31.3% T) with an overall G + C content of 38.2%. The phylogeny of Ranunculaceae based on 75 CDSs of 27 taxa showed that Ranunculoideae is paraphyletic and thus needs redefinition.

*Coptis* (Ranunculaceae) is one of the most important and well-known plant genera in eastern Asia. Dried rhizomes of *Coptis* plants are utilized for Rhizoma Coptidis (RC), a traditional Chinese medicine famous for its functions of clearing heat, dispelling dampness, and purging fire toxins (Chinese Pharmacopoeia Commission 2010). Like the other congeneric taxa, the populations of *Coptis chinensis* Franch. var. *brevisepala* W.T. Wang et Hsiao are heavily threatened or even on the verge of extinction in its natural range (Anhui, Fujian, Guangxi, Guangdong, Jiangxi, Zhejiang provinces), due to habitat destruction and long-term over-harvesting (Zhang and Zhang 2004, 2005). To better understand the evolution of this genus, we reported and characterized the first complete cp genome of *C. chinensis* var. *brevisepala*, and then presented a phylogenomic study of Ranunculaceae.

Leaf samples were collected from China, Zhejiang Province, Hangzhou City, Lin’an District, Qingliangfeng Botanical Garden. Voucher herbarium specimen (*Pan Li LP161266*) was deposited at the Herbarium of Zhejiang University (HZU). We extracted total DNA from *ca*. 3mg of the silica-gel dried leaf tissue using DNA Plantzol Reagent (Invitrogen, Carlsbad, CA), following the manufacturer’s protocol. Then, next-generation sequencing was conducted on the Illumina Hiseq Platform (Illumina, San Diego, CA). The complete cp genome was assembled via NOVOPlasty (Dierckxsens et al. 2017) with the cp genome sequence of *Coptis chinensis* (GenBank accession number: NC_036485) as a reference. The annotation was performed using Geneious 11.0.4 (Biomatters Ltd., Auckland, New Zealand). After all, clean reads were re-mapped to the draft genome and yielded the cp genome sequence of *C. chinensis* var. *brevisepala*. The complete chloroplast genome sequence was deposited in GenBank (MH509384).

The complete plastome of *Coptis chinensis* var. *brevisepala* is 155,426 bp in length, including two single copy regions (LSC: 84,488 bp and SSC: 17,402) and two inverted repeat regions (IRs: 26,768 bp). The nucleotide composition is asymmetric (30.5% A, 19.4% C, 18.7% G, and 31.3% T) with an overall G + C content of 38.2%. It contained 111 unique genes, including 78 protein-coding genes, 29 transfer RNA genes, and four ribosomal RNA genes. Sixteen genes are duplicated in the IR regions, including all four rRNA genes, seven tRNA genes, and five protein-coding genes. Each IR region contained seven tRNA genes (*trnI-CAU, trnL-CAA, trnV-GAC, trnI-GAU, trnA-UGC, trnA-ACG, trnA-GUU*). *rps19* and *ycf1* were pseudogenized due to incomplete duplication in IR regions.

The phylogeny of Ranunculaceae were reconstructed based on 75 CDSs of 26 Ranunculaceae species and one outgroup taxa (*Berberis amurensis*, Berberidaceae), using both maximum likelihood (ML) and Bayesian inference (BI) methods. ML analysis was implemented in RAxML-HPC v.8.2.10 (Stamatakis 2014) on CIPRES Science Gateway V. 3.3 (Miller et al. 2010), with the GTR + G model and 1000 bootstrap replicates. BI analysis was performed in MrBayes on XSEDE v3.2.6 (Ronquist and Huelsenbeck 2003) on CIPRES Science Gateway V. 3.3, with nst = mixed, rates = gamma, ngen =10000000, samplefreq =5000. ML and BI analyses generated the same tree topology (Figure 1). Subfamilies Hydrastidoideae, Coptidoideae, and Thalictroideae were found to be monophyletic, while subfamily Ranunculoideae was paraphyletic, because tribe Adonideae was sister to Thalictroideae. This result was consistent with the most recent phylogenetic studies on Ranunculaceae (Cossard et al. 2016; Zhang et al. 2018). Within *Coptis*, *C. chinensis* var. *brevisepala* is sister to *C. chinensis*. Overall, our data will largely enrich the genetic information of *Coptis* and facilitate future studies on its conservation genetics.

**Figure 1.**
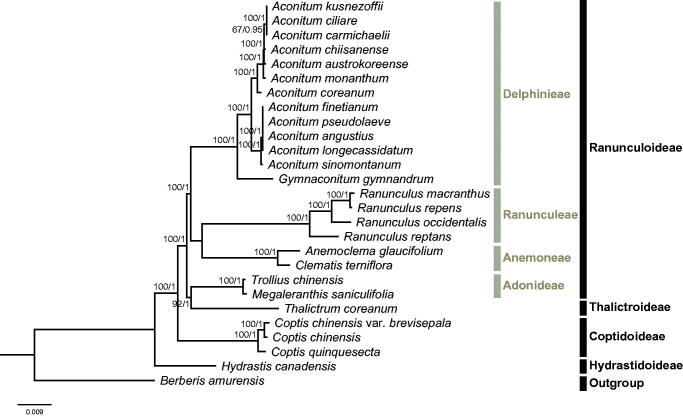
Molecular phylogeny of Ranunculaceae based on 75 CDSs of 27 taxa, with *Berberis amurensis* (Berberidaceae) as the outgroup. The accession numbers are listed as below: *Aconitum angustius* (MF155664), *Aconitum austrokoreense* (KT820663), *Aconitum carmichaelii* (KY407560), *Aconitum coreanum* (KT820667), *Aconitum chiisanense* (KT820665), *Aconitum ciliare* (KT820666), *Aconitum kusnezoffii* (KT820671), *Aconitum finetianum* (MF155665), *Aconitum longecassidatum* (KY407561), *Aconitum monanthum* (KT820672), *Aconitum pseudolaeve* (KY407562), *Aconitum sinomontanum* (MF155666), *Anemoclema glaucifolium* (NC_037194), *Berberis amurensis* (KM057374), *Clematis terniflora* (KJ956785), *Coptis chinensis* (NC_036485), *Coptis quinquesecta* (MG585353), *Gymnaconitum gymnandrum* (KT964697), *Hydrastis canadensis* (KY085918), *Megaleranthis saniculifolia* (FJ597983), *Ranunculus macranthus* (DQ359689), *Ranunculus occidentalis* (KX557270), *Ranunculus repens* (KY562594), *Ranunculus reptans* (KY562596), *Thalictrum coreanum* (KM206568), and *Trollius chinensis* (KX752098).
